# An Improvement of Survival Stratification in Glioblastoma Patients via Combining Subregional Radiomics Signatures

**DOI:** 10.3389/fnins.2021.683452

**Published:** 2021-05-13

**Authors:** Yang Yang, Yu Han, Xintao Hu, Wen Wang, Guangbin Cui, Lei Guo, Xin Zhang

**Affiliations:** ^1^School of Life Sciences, Northwestern Polytechnical University, Xi’an, China; ^2^Department of Radiology, Tangdu Hospital, The Fourth Military Medical University, Xi’an, China; ^3^School of Automation, Northwestern Polytechnical University, Xi’an, China; ^4^Institute of Medical Research, Northwestern Polytechnical University, Xi’an, China

**Keywords:** glioblastoma, multiregional, radiomics nomogram, survival stratification, magnetic resonance imaging

## Abstract

**Purpose:**

To investigate whether combining multiple radiomics signatures derived from the subregions of glioblastoma (GBM) can improve survival prediction of patients with GBM.

**Methods:**

In total, 129 patients were included in this study and split into training (*n* = 99) and test (*n* = 30) cohorts. Radiomics features were extracted from each tumor region then radiomics scores were obtained separately using least absolute shrinkage and selection operator (LASSO) COX regression. A clinical nomogram was also constructed using various clinical risk factors. Radiomics nomograms were constructed by combing a single radiomics signature from the whole tumor region with clinical risk factors or combining three radiomics signatures from three tumor subregions with clinical risk factors. The performance of these models was assessed by the discrimination, calibration and clinical usefulness metrics, and was compared with that of the clinical nomogram.

**Results:**

Incorporating the three radiomics signatures, i.e., Radscores for ET, NET, and ED, into the radiomics-based nomogram improved the performance in estimating survival (C-index: training/test cohort: 0.717/0.655) compared with that of the clinical nomogram (C-index: training/test cohort: 0.633/0.560) and that of the radiomics nomogram based on single region radiomics signatures (C-index: training/test cohort: 0.656/0.535).

**Conclusion:**

The multiregional radiomics nomogram exhibited a favorable survival stratification accuracy.

## Introduction

Glioblastoma (GBM) is the most common malignant brain tumor in adults, accounting for 15% of all brain tumors (Ostrom et al., 2015). The median overall survival (OS) of GBM is only 12 to 14 months even with aggressive therapy (van Meir et al., 2010). However, in clinical practice, the OS of patients with GBM differs significantly across patients despite having a similar pathological grade and treated with the standard Stupp protocol (Stupp et al., 2005; Omuro and DeAngelis, 2013). The wide range of the OS underscores the imperative need of individualized therapy for GBM patients. To address this, survival stratification (short- and long-term survival) is one solution as it can directly impact image-guided diagnosis and subsequent treatment options (Chen et al., 2019). In other words, the identification of effective prognostic factors of GBM patients plays an important role in delivering individualized therapies and improving patient prognosis.] The poor prognosis of GBM patients is mainly due to the heterogeneity within the individual tumors (Narang et al., 2016; Shergalis et al., 2018). This heterogeneity hampers the use of invasive biopsy-based genomic analyses, but provides an opportunity for medical imaging techniques that can view the entire tumor non-invasively and repeatably. Magnetic resonance imaging (MRI) acquires comprehensive images of the entire tumor and is a routine preoperative examination for GBM. In conventional MRI acquisition, four image sequences are commonly utilized for brain tumor diagnosis, including T1-weighted contrast-enhanced imaging (T1CE), T1-weighted imaging (T1WI), T2-weighted imaging (T2WI), and T2-weighted fluid-attenuated inversion recovery imaging (FLAIR). All of these sequences have been widely utilized in glioma diagnosis and survival analysis (Shukla et al., 2017; Zhang et al., 2020).

It is widely accepted that multiparametric MRI is pivotal for improving the efficiency of tumor diagnostic and survival stratification (Gutman et al., 2013; Huang et al., 2016; Jiang et al., 2018). On the other hand, GBM exhibits significant variations across geographical region, as has been seen on enhanced and non-enhanced MRI area. This is particularly important since different image types can have profound effects on how healthy and edema brain regions are interpreted, which can alter prognosis prediction (Gevaert et al., 2014; Jain et al., 2014; Wu et al., 2015). Thus, it is crucial for accurate diagnosis to fully utilize the heterogeneous information from each subregion within a tumor environment. To do this, radiomics leverages the correlation between underlying genetic characteristics of the tumor and the corresponding medical imaging features (Gillies et al., 2016; Kickingereder et al., 2016). By extracting high throughput quantitative imaging features, radiomics is able to analyze tumor heterogeneity non-invasively and may even correlate with clinical outcomes (Lambin et al., 2012; Aerts et al., 2014). Therefore, if the multiregional information of GBM can be captured using multi-parametric MRI and effectively exploited using radiomics, the survival stratification in GBM patients can be expected to improve. In order to solve this problem, we tried to extract features from each subregion of GBM instead of from the whole tumor volume, as is commonly performed in other studies.

As for survival analysis, a nomogram model is generally used to evaluate patient prognosis. As a statistical prediction tool, multiple factors can be incorporated into a nomogram to provide an individualized estimation of patient outcomes (Gittleman et al., 2017). Beyond traditional clinical information-based nomograms, a radiomics nomogram combining various clinical factors has great potential in GBM patient stratification (Huang et al., 2016; Kickingereder et al., 2016; Zhang et al., 2019). However, previous studies typically use a single radiomics signature that is based on features extracted from the whole tumor. Therefore, the impact of different heterogenous regions may be reduced when using such signatures. Combining multiple radiomics signatures from each subregion into one nomogram may improve the stratification performance and facilitate the effective treatment and survival of GBM patients.

Thus, the purpose of the present study was to develop and validate an effective nomogram that is based on multiregional radiomics signatures for the individualized survival stratification of GBM patients.

## Materials and Methods

### Patient Population

A total of 129 patients were retrospectively included in the current study. All patients had clinical information available from the Cancer Genome Atlas GBM Collection (TCGA-GBM^1^) and corresponding multimodal MRI data available from the Cancer Imaging Archive (TCIA^2^). The complete radiological data of the TCIA-GBM consists of 262 multimodal MRI scans obtained from eight institutions (Clark et al., 2013). The data used in the present study included the pre-operative baseline scans acquired with T1WI, T1CE, T2WI, and FLAIR sequences. The patients were assigned to two cohorts: a training cohort comprising 99 patients from institutions 2, 6, 8, and 12, and an independent test cohort comprising 30 patients from institutions 14, 19, 27, and 76. No institutional review board approval was required since TCGA is a publicly available dataset without patient identifiers. Except for the multi-parametric MRI data, the clinicopathologic information, including gender, age, KPS, prognostic treatment, TP53, PTEN, EGFR, and IDH1, were obtained from TCGA GBM Project for all patients.

### Image Preprocessing and Tumor Segmentation

The pre-processed and labeled MRI data are available through TCIA (Bakas et al., 2017). The pre-operative MRI volumes were initially co-registered to the T1WI and then skull-stripped. Consistent with the BRAin Tumor Segmentation (BRATS) challenge, the segmentation labels delineate three parts of each tumor, including enhanced (ET) and non-enhanced tumor (NET) regions, as well as the peritumoral edema (ED). Tumor segmentation was achieved by integrating an automated pre-segmentation process with manual corrections from a board-certified neuroradiologist. The automated hybrid generative-discriminative method, which won first place at the International Multimodal BRATS 2015, was applied to produce a set of labels. Then, these automatically segmented labels were modified and the misclassified labels were manually corrected.

### Radiomics Feature Extraction and Signature Construction

A variety of imaging features were extracted from the MRI datasets, including spatial information, intensity, volumetric, morphologic, histogram, and textural parameters. A total of 537 radiomics features, including 179 features from ET, NET or ED, were extracted for each subject. A detailed description of the extracted spatial features can be found in Supplementary Table 1.

The number of subjects (independent samples) should exceed the number of selected features by a factor of at least 10 according to the guideline (Harrell, 2015). Thus, the combination of the least absolute shrinkage and selection operator (LASSO) algorithm and Cox survival model was used to select the most useful prognostic features in the training dataset. The LASSO method utilizes a regularization parameter λ to shrink the coefficients of all irrelevant features to zero. Here, λ was optimized to maximize the area under the receiver operating curve (AUROC) in a 10-fold cross-validation procedure. Then, the Cox regression model was constructed from the features with non-zero coefficients. After that, radiomics scores (Radscores) were computed for each patient through a linear combination of the selected LASSO features, weighted by their respective coefficients. These scores can be useful by further stratifying the GBM patients.

Specifically, when considering the influence of different tumor regions, two kinds of feature selection strategies were applied and compared in this study:

(i) All 537 features from the ET, NET, and ED were combined as the input to the model, and eventually one single Radscore was obtained and analyzed (denoted as Radscore-Con);
(ii) The features from the ET, NET, and ED regions were fed into the model separately to produce three independent Radscores (denoted as Radscore-ET, Radscore-NET and Radscore-ED) for the following survival analysis.

### Validation of Radiomics Signatures

By using Kaplan-Meier survival analysis, the potential correlation between radiomics signatures and OS was assessed in the training cohort and validated in the test cohort. The optimal cutoff values of Radscores were selected based on their association with the patients’ OS in the training cohort using the maximally selected rank statistics from the “maxstat” R package (Delgado et al., 2014). Then, the same cutoff values were applied to the test cohorts. Patients were classified into high- and low-Radscore groups accordingly for further analysis. Evaluation of the radiomics signature built from different features was performed by adding the Radscore as an independent factor in the multivariable Cox proportional hazard model (backward step-down selection; the Akaike information criterion, AIC). This model also integrated the general clinical risk factors as well.

### Radiomics Signature Assessment

Radiomics and clinical nomograms were both applied to the training cohort based on the multivariate Cox analysis to indicate the increased predictive value of the radiomics signatures to the clinical risk factors for individualize OS assessment. Specifically, the radiomics nomograms were generated by integrating the radiomics signatures and clinical risk factors, while the clinical nomogram contained only the clinical risk factors. Then, the increased predictive value of the radiomics signatures relative to clinical risk factors was assessed by calculating the discrimination, reclassification, calibration, and clinical usefulness. The effect of the radiomics signatures was compared with that of the clinical nomogram. Before this, two radiomics nomograms corresponding to two feature selection strategies were also constructed and compared.

For model fitting assessment, AIC was calculated to assess the risk of model overfitting. And the performance of different models was evaluated using the integrated Brier score (IBS) by calculating the prediction error over time and the compared, which represents the weighted average squared distance between the predicted probability of the established model and the observed survival status. IBS values can range from 0, i.e., a perfect model, to 0.25, i.e., a non-informative model with a 50% survival/non-survival prediction.

For discrimination performance assessment, calibration curves were derived to compare the consistency between the OS predicted by the radiomics and clinical nomograms and the actual OS. The Harrell concordance index (C-index) was calculated to quantify the discrimination performance. Meanwhile, decision curve analysis (DCA) enabled the determination of clinical usefulness of the radiomics nomograms by measuring the net benefit at several probability thresholds.

### Statistical Analysis

Statistical analysis performed using the R software. R packages that were utilized are summarized as follows: the “surv_cutpoint” function of “survminer” package was applied for cutoff point calculation; the “glmnet” and “survival” packages were applied for LASSO Cox regression; the “rms” package was applied for multivariate Cox regression, calibration plots and nomograms; the “Hmisc” package was applied for C-indexes comparisons; the “rmda” package was applied for DCA; and the “ipred” package was applied for IBS calculation.

## Results

### Patient Characteristics and Radiomics Signature Construction

Clinical characteristics of all patients are shown in Supplementary Table 2. We used the LASSO Cox regression model to select features and build prognostic classifiers from these features. Ten potential predictors were identified from the 179 features for each subregion, i.e., ET, NET, and ED, in the training cohort Eight potential predictors were identified when all the features were concatenated together. The radiomics signatures were then constructed with weights applied to the coefficients in each model and the Radscores were obtained. The relevant equations for this process can be found in Supplementary Table 3.

**FIGURE 1:**
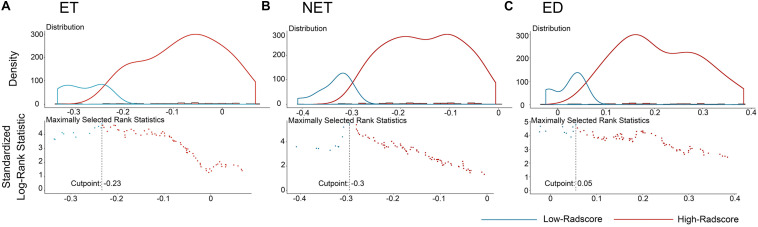
The optimum cutoff score calculation of the Radscores. **(A)** The cutoff plot for Radscores of ET, **(B)** the cutoff plot for Radscores of NET, and **(C)** the cutoff plot for Radscores of ED. The low-radscores are indicated in blue and high-radscores are indicated in red.

**FIGURE 2:**
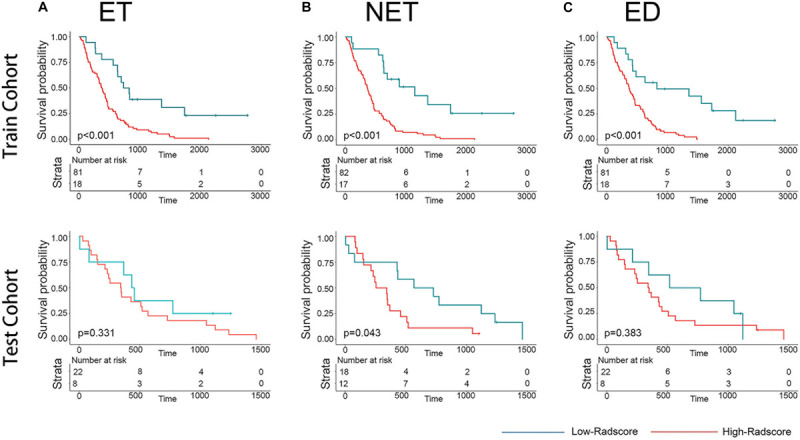
Graphs show results of Kaplan-Meier survival analysis according to the radiomics signature of ET **(A)**, NET **(B)**, and ED **(C)** for patients in the training cohort (first row) and those in the test cohort (second row). The low-radscores are indicated in blue and high-radscores are indicated in red.

The distributions of the Radscores of different sub-regions in the training cohort are shown in Figure 1. The optimal cutoff value was −0.233, −0.295, and 0.055 for the Radscore-ET, Radscore-NET and Radscore-ED, respectively, and −0.321 for the Radscore-Con. Accordingly, patients were stratified into a low-Radscore group and high-Radscore group. The OS rate in the high-Radscore group and low-Radscore group in the training and test cohorts are provided in Supplementary Table 4. Combined with Figure 2, we found that patients with lower Radscores generally exhibited a better OS. This finding was assessed in the training cohort and then confirmed in the test cohort.

### Assessment of Radiomics Signatures

**FIGURE 3:**
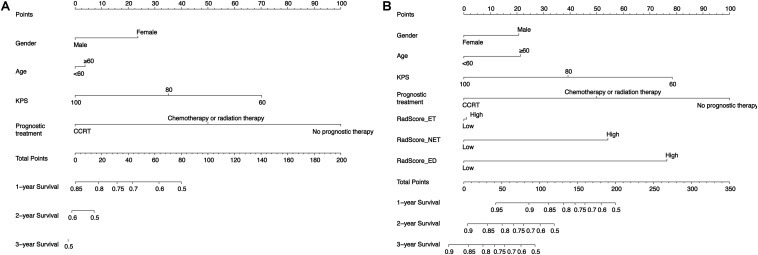
Use of the constructed clinical nomogram **(A)** and the radiomics nomogram **(B)**.

To validate the radiomics signatures and to provide a quantitative and clinical method to predict the probability of the 1-, 3-, and 5-year OS of GBM patients, a clinical nomogram and two radiomics nomograms were constructed based on the training cohort data (Figure 3). Four clinical risk factors, including days to birth, KPS, prognostic treatment and spatial_frontal, were significantly associated with survival and were included in the models (Table 1).

**TABLE 1:** Clinical factors used for overall survival (OS) stratification of glioblastoma patients.

	**Univariate cox**		**Multivariate cox**	
	**regression**		**regression**	
	**HR (95% CI for HR)**	***P* value**	**HR (95% CI for HR)**	***P* value**
Gender	0.89 (0.58–1.4)	0.580	0.65 (0.38–1.11)	0.115
Age	1.3 (0.81–2.10)	0.275	1.07 (0.65–1.76)	0.789
KPS	0.97 (0.95–0.99)	0.004*	0.97 (0.95–0.99)	0.005*
Prognostic treatment	3.6 (2.3–5.6)	<0.001*	2.48 (1.30–4.75)	0.006*
TP53	0.89 (0.53–1.5)	0.64	NA	
PTEN	0.68 (0.39–1.2)	0.16	NA	
EGFR	0.88 (0.46–1.7)	0.71	NA	
IDH1	0.3 (0.041–2.1)	0.23	NA	
SPATIAL_Frontal	1 (0.99–1)	0.179	NA	
SPATIAL_Temporal	1 (1–1)	0.51	NA	
SPATIAL_Parietal	1 (1–1)	0.20	NA	
SPATIAL_Basal_G	1 (0.98–1)	0.56	NA	
SPATIAL_Insula	1 (0.97–1.1)	0.52	NA	
SPATIAL_CC_Fornix	1 (0.95–1.1)	0.63	NA	
SPATIAL_Occipital	1 (0.99–1)	0.52	NA	
SPATIAL_Brain_stem	1 (0.99–1.1)	0.14	NA	

*P < 0.05.

As for the radiomics nomograms, we found that the discrimination performance of the one constructed from all the independent RadScore risk factors, including Radscore-ET, Radscore-NET, and Radscore-ED (C-index/AIC: training cohort: 0.717/452; test cohort: 0.655/143) was better than the nomogram constructed from the Radscore-Con as a single risk factor (C-index/AIC: training cohort: 0.656/468; test cohort: 0.535/146). Thus, the nomogram constructed from the combination of the Radscore-ET, Radscore-NET, and Radscore-ED signatures was used for further analysis.

**FIGURE 4:**
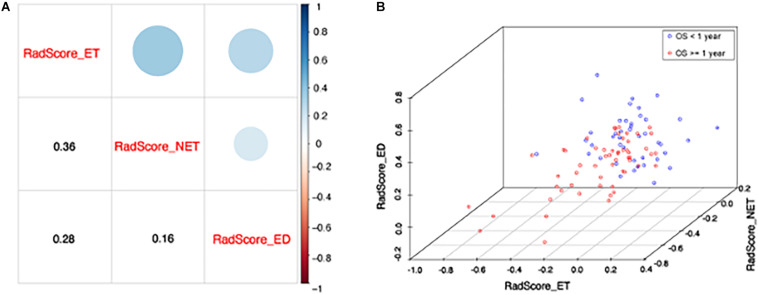
**(A)** The correlation between each RadScore. The lower corner shows the Pearson correlation coefficients and upper corner indicates the correlation degree. **(B)** The scatter plot shows patients with short (<1 year) and long survival (≥1 year). X, y, and z axis represents RadScore-ET, RadScore-NET, and RadScore-ED, respectively.

Meanwhile, the correlation between the subregion RadScores was examined to test whether overfitting exist. The correlation coefficients between RadScore-ET and Radscore-NET, between Radscore-ET and Radscore-ED, and between Radscore-NET and Radscore-ED were 0.36, 0.28, and 0.16, respectively (Figure 4A). Figure 4B indicates the scatter plot based on these three RadScores and 1-year survival difference between two groups can be observed obviously.

**FIGURE 5:**
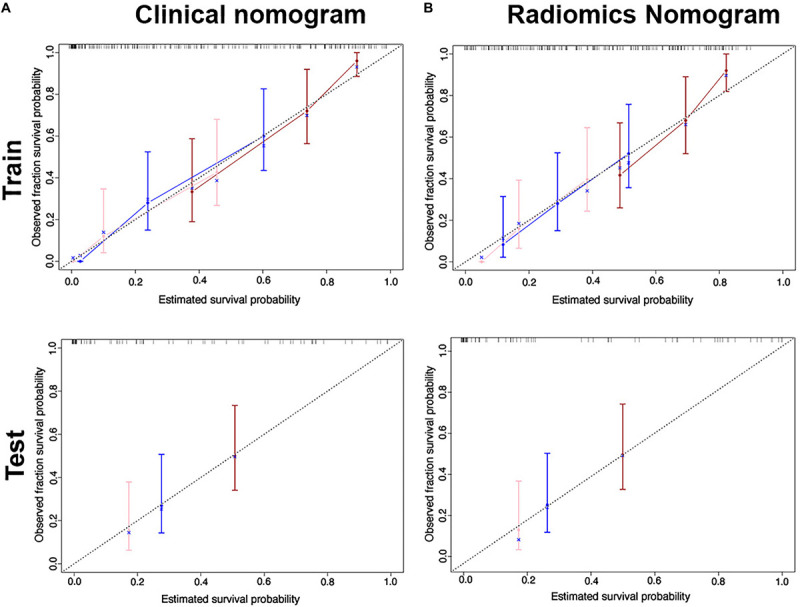
Calibration curves for the clinical nomogram **(A)** and radiomics nomogram **(B)** show the calibration of each model in terms of the agreement between the estimated and observed 1-, 2- and 3-year outcomes for the training cohort (first row) and the test cohort (second row).

We found good agreement between three actual survival observations and the survival estimates determined by the radiomics nomograms at 1, 3, and 5 years in both training and test cohorts, as is depicted in the calibration curves of the nomograms (Figure 5). The C-index, IBS and AIC estimations for the clinical and radiomics nomogram models are also summarized in Table 2. As assessed with the IBS (training cohort/test cohort: 0.077/0.066, lower values indication better model performance), the C-index (training cohort/test cohort: 0.717/0.655, higher values indication better discriminative ability) and AIC (training cohort/test cohort: 452/143, lower values indicating better model performance), the radiomics nomogram performed better than the clinical nomogram in both the training and test cohorts.

**TABLE 2:** Performance of models.

**Model**	**Training cohort**				**Test cohort**			
	**C-Index**	**Concordance probability**	**IBS**	**AIC**	**C-Index**	**Concordance probability**	**IBS**	**AIC**
Clinical nomogram	0.633	0.040	0.094	470	0.560	0.051	0.127	112
Radiomics_ET signature	0.632	0.034	0.096	673	0.535	0.054	0.151	145
Radiomics_NET signature	0.632	0.034	0.093	678	0.584	0.061	0.134	142
Radiomics_ED signature	0.654	0.031	0.091	666	0.557	0.052	0.146	146
Radiomics_Con Nomogram	0.656	0.040	0.090	468	0.535	0.053	0.127	146
Radiomics_ET+Radiomics_ NET + Radiomics_ED Nomogram	0.717	0.038	0.077	452	0.655	0.066	0.125	143

Meanwhile, multivariable Cox regression analysis after adjustment for clinical factors showed that the Radscores were an independent and powerful prognostic factor for OS in the training and test cohorts (Supplementary Figure 1). The *p*-values for the Radscore-ET, Radscore-NET, and Radscore-ED were 0.96, 0.002, and 0.003, respectively. Among these, the Radscore-ED had the highest hazard ratio and played an important role in prognosis.

### Clinical Use

**FIGURE 6:**
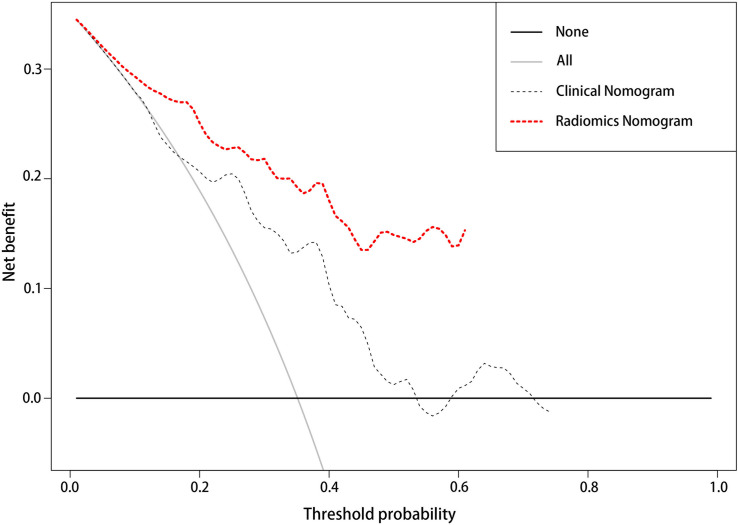
Decision curve analysis for each model.

The DCA (Figure 6) showed that in most of the reasonable threshold probabilities, the net benefit of the radiomics nomogram was slightly higher than that of the clinical nomogram.

## Discussion

The current study investigated the influence of a multiregional radiomics nomogram in the survival stratification of GBM patients. We found an optimal radiomics nomogram to be one that integrated three radiomics signatures from different tumor subregions and clinical risk factors. This optimal nomogram outperformed the one built only by clinical risk factors as well as the one constructed from the radiomics signature from the entire tumor, as is commonly used in previous works.

Clinicopathological risk factors such as KPS, gender, age, IDH status and O6-methylguanine-DNA methyltransferase (MGMT) have been the most utilized factors used to construct survival nomograms of GBM patients in previous studies. Using these risk factors, various achievements have been obtained, however, due to the lack of MGMT information for most of the patients from TCGA-GBM, this factor was not included in our study. Based on the univariate logistic regression used here, we found that various clinical risk factors, including days to birth, KPS, prognostic treatment and spatial_frontal were significantly associated with survival. These factors were therefore chosen to further construct the clinical nomogram. Using this clinical nomogram, we observed a similar survival stratification performance as has been reported throughout literature.

Consistent with previous studies focusing on radiomics nomograms, we observed a performance improvement after adding the radiomics signature, suggesting that radiomics signatures were more robust compared with clinical risk factors (Zhang et al., 2019). However, the heterogeneity pattern of ET, NET, and ED are different and may provide specifically heterogenous information. Thus, instead of extracting imaging features from the whole tumor area or only the contrast-enhanced area of glioblastoma, we further analyzed information from different heterogeneous areas of the tumor environment. This strategy takes advantage of each sub-region into account and evaluates the significance of each. The results further indicated that the nomogram that utilized three radiomics signatures as separate risk factors performed better than the nomogram based on a single radiomics signature. Along these lines, the C-index improved from 0.656 to 0.717 in the training cohort and from 0.535 to 0.655 in the test cohort using these two model types.

As for each tumor region, the Radscore-ED signature derived from the peritumoral edema region outperformed those from the other two regions, i.e., showed the highest predictive performance. It has been reported in multiple previous studies that the heterogeneity of GBM in not limited to the tumor margins and that the peritumoral brain parenchymal zone (PBZ) is also involved. In fact, about 90% of GBM recurrences occur in the PBZ. The microenvironment of GBM-PBZ suggests that the interaction of specific cells (microglial cells, glioma cells, neuroglial, and vascular endothelial) and molecular events in PBZ leads to micro-vascularity, tumor infiltration, and compromise of the blood brain barrier. This process eventually contributes to poor survival of GBM patients; however, few studies have investigated the PBZ. Thus, the prognostic influence of ED in GBM patients’ needs to be demonstrated in future studies.

The data used in this study was obtained from different institutions and acquired on different MRI scanners. This combined data was further split into a training and test cohort, where the test cohort was completely independent. These factors in general make our study more robust than previous studies; however, several limitations still exist for the current work. Since the data were collected from a public database, we only included images with conventional sequences in our radiomics signatures. Radiomics features derived from functional or more complex MR sequences, such as diffusion or perfusion sequences, were therefore not investigated. Similarly, genetic information was often incompletely provided by the public databased and was also excluded from this work.

In conclusion, in this study, a radiomics nomogram model based on multiple radiomics signatures was developed and validated in GBM patients. This model leveraged multi-institutional and multi-parametric data collected from TCGA for enhanced survival stratification of GBM patients compared to traditional nomograms. The encouraging predictive accuracy and survival stratification performance of the proposed multiregional radiomics nomogram demonstrated great potential for clinical applications.

## Data Availability Statement

The datasets presented in this study can be found in online repositories. The names of the repository/repositories and accession number(s) can be found below: https://wiki.cancerimagingarchive.net/display/Public/TCGA-GBM. The clinical and genetic data can be found in: https://portal.gdc.cancer.gov/.

## Ethics Statement

Ethical review and approval was not required for the study on human participants in accordance with the local legislation and institutional requirements. Written informed consent for participation was not required for this study in accordance with the national legislation and the institutional requirements.

## Author Contributions

YY and YH performed the data collection or acquisition, statistical analysis. All authors contributed to the conception and study design, interpretation of the results, drafting the manuscript work or revising it critically for important intellectual content and approval of final version to be published and agreement to be accountable for the integrity and accuracy of all aspects of the work.

## Conflict of Interest

The authors declare that the research was conducted in the absence of any commercial or financial relationships that could be construed as a potential conflict of interest.

## Footnotes

1 cancergenome.nih.gov

2 www.cancerimagingarchive.net
