# Supplementary material for: An Improvement of Survival Stratification in Glioblastoma Patients via Combining Subregional Radiomics Signatures
Source: Front Neurosci. 2021 May 13;15:683452. doi: 10.3389/fnins.2021.683452 (PMC8161502; doi:10.3389/fnins.2021.683452)
Supplement: Supplementary file 1 [file Data_Sheet_1.docx]

**Table S1. Detailed Description of the Clinical Spatial Features**

| **Feature ID** | **Description** |
| --- | --- |
| SPATIAL_Frontal | Percentage of TC volume in the frontal lobe. |
| SPATIAL_Temporal | Percentage of TC volume in the temporal lobe. |
| SPATIAL_Parietal | Percentage of TC volume in the parietal lobe. |
| SPATIAL_Basal_G | Percentage of TC volume in the regions of putamen, caudate nucleus, globus pallidus, subthalamic nucleus, nucleus accumbens, internal capsule, and thalamus. |
| SPATIAL_Insula | Percentage of TC volume in the region of the insula. |
| SPATIAL_CC_Fornix | Percentage of TC volume in the regions of corpus callosum, fornix, and cingulate. |
| SPATIAL_Occipital | Percentage of TC volume in the occipital lobe. |
| SPATIAL_Cere | Percentage of TC volume in the region of the cerebellum. |

Note: TC: Tumor core

**Table S2. Demographic and Clinical Characteristics of GBM Patients in the Training and Test Cohort**

| **Characteristic** | **Training Cohort** | **Test Cohort** |
| --- | --- | --- |
| **Gender** Male | 35 (35.35) | 13 (43.33) |
| Female | 64 (64.65) | 17 (56.67) |
| **KPS^*^** | 80 (60-100) | 80 (40-80) |
| **Age (years old) ^†^** | 50.8 (48.2-66.3) | 55.2 (59.4-68.6) |
| **Follow-up time (days)^†^** | 384 (183-630) | 348 (151.5-531) |
| **IDH** Mutant | 2 (2.02) | 1 (3.45) |
| Wild | 97 (97.98) | 29 (96.67) |
| **TP53** Mutant | 20 (20.20) | 7 (23.33) |
| Wild | 79 (79.80) | 23 (76.67) |
| **PTEN** Mutant | 17 (17.18) | 10 (33.33) |
| Wild | 82 (82.82) | 20 (66.67) |
| **EGFR** Mutant | 11 (11.11) | 5 (16.67) |
| Wild | 88 (88.89) | 25 (83.33) |
| **Spatial in Frontal^†^** | 5.69 (0 – 27.21) | 9.27 (0-62.08) |
| **Spatial in Temporal^†^** | 36.20 (0.10 – 83.55) | 34.60 (0.51-72.45) |
| **Spatial in Parietal^†^** | 1.43 (0 – 32.28) | 4.51 (0-21.50) |
| **Spatial in Basal G^†^** | 0.08 (0 – 5.09) | 0.72 (0-10.66) |
| **Spatial in Insula^†^** | 0.47 (0 – 2.58) | 0.23 (0-5.70) |
| **Spatial in CC fornix^†^** | 0 (0 – 0.01) | 0 (0-1.48) |
| **Spatial in Occipital^†^** | 0 (0 – 0.03) | 0 (0-0.09) |
| **Spatial in Brain stem** | 0 (0-0) | 0 (0-0.10) |

Note. Unless otherwise specified, data are numbers of patients, with percentages in parentheses. No difference was found between the training and the test data set for either the clinical characteristics or the follow-up data.

* Data in parentheses are ranges.

† Data in parentheses are interquartile range

**Table S3. Radiomics signature calculation formula**

| Radscore-ET = 0.219454820 ∙ SOLIDITY_ET + 0.1854476053 ∙ ECCENTRICITY_ET + (-0.0016264664) ∙ INTENSITY_Mean_ET_T2 + 4.450693e^-04^ ∙ HISTO_ET_T1_Bin6 + (-1.206945e^-02^) ∙ HISTO_ET_T1_Bin9 + (-2.953768e^-02^) ∙ HISTO_ET_T1_Bin10 + (-6.574296e^-01^) ∙ TEXTURE_GLRLM_ET_T2_LGRE + (-2.198876e^01^) ∙ TEXTURE_GLSZM_ET_T1_SZLGE + 2.975086e^-03^ ∙ TEXTURE_GLSZM_ET_T2_LZLGE + 3.520358e^-04^ ∙ TEXTURE_GLSZM_ET_FLAIR_LZE |
| --- |
| Radscore-NET = (-0.3689748) ∙ VOLUME_NET_OVER_WT + (-0.4749949) ∙ SOLIDITY_NET + 6.426720e^-03^ ∙ HISTO_NET_T2_Bin2  + 2.103468e^-03^ ∙ HISTO_NET_T2_Bin4 + (-2.284474e^-03^) ∙ HISTO_NET_T2_Bin7 + (-1.384110e^-03^)$\cdot$HISTO_NET_FLAIR_Bin2 + 6.883969e^-04^$\cdot$HISTO_NET_FLAIR_Bin7 + (-7.152464e^-01^)$\cdot$ TEXTURE_GLRLM_NET_T2_LRLGE + 2.5411909516$\cdot$ TEXTURE_GLSZM_NET_FLAIR_SZLGE + 1.467716e^-06^$\cdot$TEXTURE_NGTDM_NET_T1_Complexity |
| Radscore-ED = (-0.1652031)$\cdot$ SOLIDITY_ED + (-0.07528354) $\cdot$ HISTO_ED_T1_Bin1 + (-0.01584542)$\cdot HISTO\_ED\_T1\_Bin10$ + 0.0010044853$\cdot$HISTO_ED_T2_Bin2 + (-2.397592e^-03^) $\cdot$ HISTO_ED_T2_Bin7 + (-0.0034031282) $\cdot$ HISTO_ED_T2_Bin8 + (-2.967122e^-02^) $\cdot$ TEXTURE_GLRLM_ED_FLAIR_LRLGE + 3.893689e^-01^ $\cdot$ TEXTURE_GLSZM_ED_T1_SZE + (-3.648048e^-03^) $\cdot$ TEXTURE_GLSZM_ED_T1_LZLGE + 9.812213e^-02^ $\cdot$ TEXTURE_NGTDM_ED_T2_Busyness |
| Radscore-Con = (-3.822443e^-03^) $\cdot$ HISTO_ET_T1_Bin9 + (-1.506377e^-02^) $\cdot$ HISTO_ET_T1_Bin10 + (-6.140192e^-02^) $\cdot$ VOLUME_NET_OVER_WT + (-4.786771e^-01^) $\cdot$ SOLIDITY_NET + 1.832719e^-03^ $\cdot$ HISTO_NET_T2_Bin2 + (-4.384262e^-04^) $\cdot$ HISTO_NET_T2_Bin7 + (-1.949495e^-03^) $\cdot$ HISTO_NET_FLAIR_Bin1 + (-2.876273e^-03^) $\cdot$ SOLIDITY_ED |

**Table S4. Overall survival rate in high- and low-Radscore Groups**

| **Parameter** | | **Training Cohort** | | **Test Cohort** | |
| --- | --- | --- | --- | --- | --- |
|  |  | **high-Radscore Group** | **low-Radscore Group** | **high-Radscore Group** | **low-Radscore Group** |
| **Radscore-ET** | No. of patients | 81 (81.8) | 18 (18.2) | 22 (73.3) | 8 (26.7) |
|  | Median (day)* | 372 (175-600) | 775 (576-1256.5) | 344 (168-531) | 444.5 (296.5-854.25) |
|  | Shortest (day) | 16 | 111 | 30 | 5 |
| **Radscore-NET** | No. of patients | 82 (82.8) | 17 (17.2) | 18 (60.0) | 12 (40.0) |
|  | Median (day)* | 366 (179.25-588.5) | 873 (630-1356) | 298 (168-433.25) | 647 (340.25-1149) |
|  | Shortest (day) | 16 | 103 | 82 | 5 |
| **Radscore-ED** | No. of patients | 81 (81.8) | 18 (18.2) | 22 (73.3) | 8 (26.7) |
|  | Median (day)* | 372 (175-621) | 795 (425.5-1688.5) | 346 (148.5-494.75) | 645.5 (312.75-1061.25) |
|  | Shortest (day) | 16 | 123 | 30 | 5 |
| **Radscore-Con** | No. of patients | 85 (85.9) | 14 (14.1) | 21 | 9 |
|  | Median (day)* | 382 (178-621) | 881 (582.25-1302.75) | 342 (153-567) | 434 (368-772) |
|  | Shortest (day) | 16 | 127 | 30 | 5 |

Note. Unless otherwise specified, data in parentheses are percentages.

* Data in parentheses are interquartile ranges.


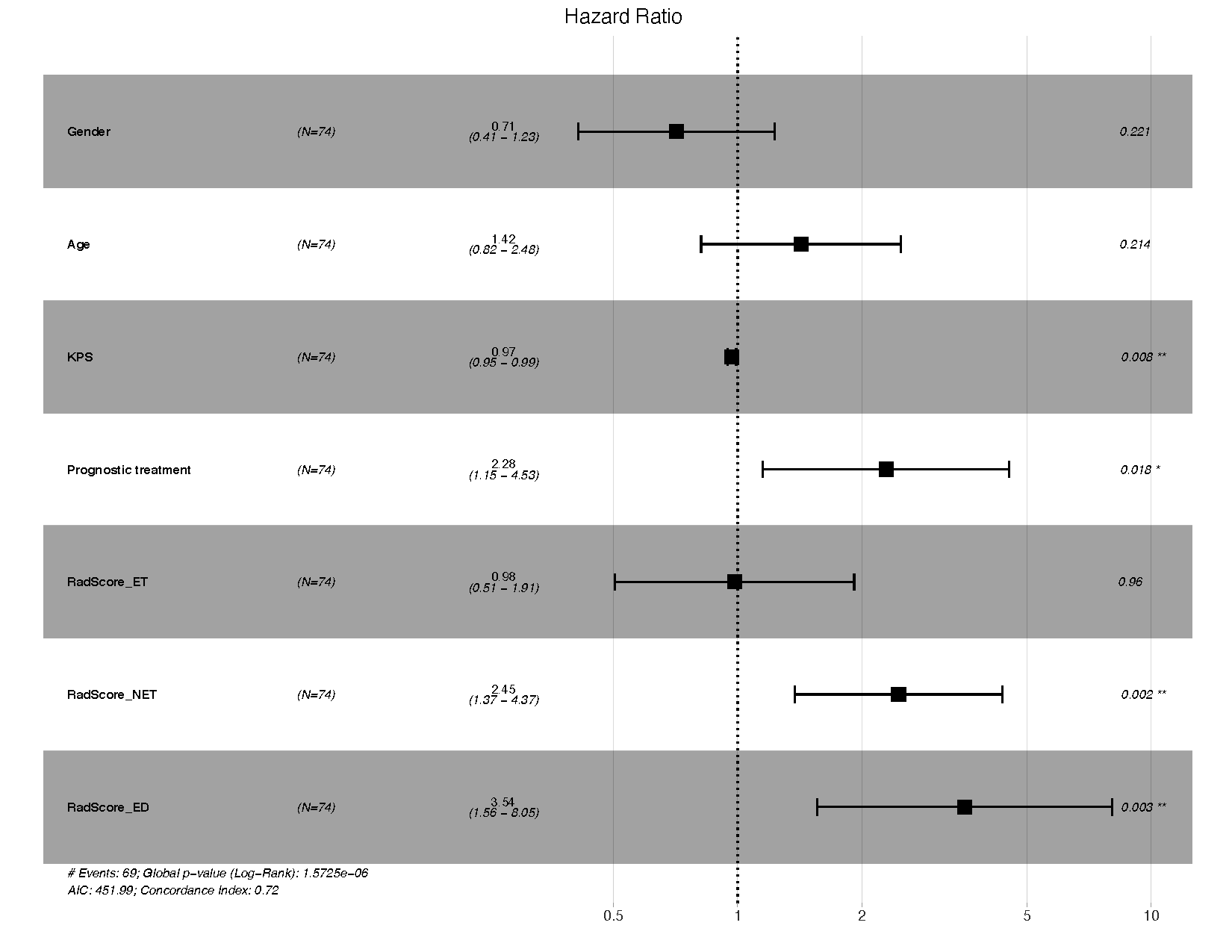


Figure S1. Forest plot of independent predictors evaluated with a multivariate regression model.
